# Cefquinome-loaded microsphere formulations against *Klebsiella pneumonia* infection during experimental infections

**DOI:** 10.1080/10717544.2018.1461958

**Published:** 2018-04-13

**Authors:** Shaoqi Qu, Cunchun Dai, Jiajia Zhu, Li Zhao, Yuwen Li, Zhihui Hao

**Affiliations:** a Agricultural Bio-pharmaceutical Laboratory, Qingdao Agricultural University, Qingdao, China;; b National-Local Joint Engineering Laboratory, Agricultural Bio-pharmaceutical Technology, Qingdao, China

**Keywords:** Two-fluid nozzle spray drier, polylactic acid microspheres, *Klebsiella pneumonia*, cefquinome, pharmacodynamics

## Abstract

The aim of this study was to prepare cefquinome-loaded polylactic acid microspheres and to evaluate their *in vitro* and *in vivo* characteristics and pharmacodynamics for the therapy of pneumonia in a rat model. Microspheres were prepared using a 0.7 mm two-fluid nozzle spray drier in one step resulting in spherical and smooth microspheres of uniform size (9.8 ± 3.6 μm). The encapsulation efficiency and drug loading of cefquinome were 91.6 ± 2.6% and 18.7 ± 1.2%, respectively. *In vitro* release of cefquinome from the microspheres was sustained for 36 h. Cefquinome-loaded polylactic acid microspheres as a drug delivery system was successful for clearing experimental *Klebsiella pneumonia* lung infections. A decrease in inflammatory cells and an inhibition of inflammatory cytokines TNF-α, IL-1β and IL-8 after microspheres treatment was found. Changes in cytokine levels and types are secondary manifestations of drug bactericidal effects. Rats were considered to be microbiologically cured because the bacterial load was less than 100 CFU/g. These results also indicated that the spray-drying method of loading therapeutic drug into polylactic acid microspheres is a straightforward and safe method for lung-targeting therapy in animals.

## 1. Introduction

*Klebsiella (K.) pneumonia* is an opportunistic pathogen causing life-threatening nosocomial infections and one of the most frequently isolated Gram-negative bacterial pathogens. The rapidly progressive clinical course of *K. pneumonia* is often complicated by lung multilobe involvement and abscesses. The high mortality of these infections often leaves little time to initiate effective antibiotic treatment (Yoshida et al., 2001).

Current treatments for pneumonia involves the long-term oral administration of high doses of multiple drugs (Bramwell & Williams, 2009). This treatment can lead to serious side effects including liver or kidney damage. Selectivity of a drug distribution to lungs cannot be ensured by an oral route and the difficulty of delivering drugs to sites deep within the lungs, to the actual site of the infection. Targeting drug delivery system increases the drug quantity at the target site while simultaneously decreasing the distribution to other body regions (Liu et al., 2017). Lung delivery of drugs is a major clinical problem (Suarez et al., 2001; Zhou et al., 2005; Hirot et al., 2007).

Microsphere drug delivery systems (or microspheres) are an alternative to uncomplexed drug delivery and provide a slower intracellularly release of therapeutic drug doses (Liu et al., 2003). Biodegradable polymers such as polylactic acid (PLA), polylactic-co-glycolic acid (PLGA) and albumin have been used for microsphere encapsulation matrices and are biologically inert and nontoxic (Okada et al., 1995; Qu et al., 2017). The rate and extent of drug release from microspheres is dependent upon the particle size, extent of crosslinking as well as the physiochemical properties of the drug (Deng et al., 1999).

Spray-drying is a single step process applicable to even heat-sensitive materials (Zhang et al., 2000). This technique has a potential for the large scale encapsulation of drugs due to the use of mild conditions. Furthermore, it generally yields microparticles of a narrow size distribution that possess high encapsulation efficiencies (Palazzo et al., 2013).

Cefquinome (CEQ) is an aminothiazolyl cephalosporin and the first member of the fourth-generation cephalosporins developed for use in veterinary medicine (Kasravi et al., 2011; Xiao et al., 2013). This drug has demonstrated *in vitro* and *in vivo* efficacy against a wide range of Gram-negative and Gram-positive bacteria (Widmer et al., 2009). Pneumonia caused by *K. pneumonia* results in the recruitment and activation of leucocytes that involves coordinated expression of both pro- and anti-inflammatory cytokines (Song et al., 2004). For example, the IL-10 produced during *K. pneumonia* lung infection promotes bacterial survival. Inhibition of its anti-inflammatory properties promotes bacterial killing because pro-inflammatory cytokines such as IL-1β, IL-6 and TNF-α are allowed to be activated (Greenberger et al., 1995).

In the current study, we prepared PLA microspheres as a CEQ carrier in a single step using a 0.7 mm two-fluid nozzle spray drier and were characterized in terms of morphology, size, drug-loading coefficient, encapsulation ratio and *in vitro* performance. We then evaluated the pharmacodynamics of the coupled microspheres in the lungs of rats infected with *K. pneumonia*.

## 2. Materials and methods

### 2.1. Materials

Polylactic acid (inherent viscosity 0.28 dL/g, MW 30 kDa) was produced by Shandong Institute of Medical Instruments (Jinan, China). Cefquinome injection (2.5%, wt/v) was purchased from MSD Animal Health (Dublin, Ireland). Acetonitrile (HPLC grade) was purchased from Fisher Scientific (Pittsburgh, PA, USA). Purified water was produced using a MilliQ Plus water filtration system. All other chemicals and solvents used were purchased from Sinopharm Chemical Reagent (Shanghai, China) and were of analytical grade or better.

### 2.2. Preparation of the microspheres

CEQ-loaded microspheres were prepared at the theoretical loading of 20% (20 mg of CEQ per 80 mg of polymer) by spray-drying a solution in a Lab Spray-Dryer SY-6000 (Shanghai, China). The dispersion was prepared dispersing 200 mg of CEQ powder in 50 mL methylene chloride containing 800 mg of PLA under gentle vortexing. Spray drying was performed with the following process parameters: inlet temperature, 50 °C; supply rate for solution, 8 mL/min; spray-flow 700 NL/h.

### 2.3. Microsphere characterization

The morphology of the microspheres was evaluated by scanning electron microscopy (SEM; Jeol, JSM-7500F, Tokyo, Japan). Dry microspheres were mounted on carbon-taped copper stubs and sputter coated with gold for 1.5 min at 8 mA for observation.

Five hundred particles were measured for particle size distribution and measurements were carried out using SEM. The amount of CEQ encapsulated in the PLA microspheres was determined by a high-performance liquid chromatography (HPLC) method. Briefly, 50 mg of CEQ-loaded PLA microspheres were dissolved in 50 mL of methylene chloride followed by a 10-fold dilution with the HPLC mobile phase consisting of acetonitrile–water (85:15, v/v). After centrifugation at 6000×*g* for 10 min, the supernatant was collected and used for injection into an Agilent 1200 HPLC system (Agilent Technologies, Santa Clara, CA) to quantify CEQ. All measurements were conducted in triplicate and the results were expressed as mean ± SD.

CEQ loading was expressed as mg of encapsulated CEQ per 100 mg of microspheres. CEQ entrapment efficiency (%) was calculated as follows: amount of CEQ in microspheres / CEQ quantity added in the microsphere preparation process.

### 2.4. *In vitro* release study

CEQ-loaded PLA microspheres (50 mg) were dispersed into 2 mL of phosphate buffer saline (PBS) (pH 7.4) and membrane dialyzed (MW cutoff 8-14 kDa, Millipore, MA, USA) against 250 mL of PBS (pH 7.4). An identical amount of solid CEQ was used as a control. The system was agitated at 37 °C ± 1 °C in a shaking water bath set at 100 rpm for 48 h in the dark. At predetermined time intervals, medium was withdrawn in 2 mL volumes and replaced with an equal volume of fresh release medium. The samples were filtered and the CEQ content were determined by HPLC as described above. The release studies were performed in triplicate and the results are presented as the mean ± SD. The results of all measurements were used to calculate cumulative drug release.

### 2.5. *In vivo* of pharmacodynamics studies

#### 2.5.1. Pneumonia model in rats

Pulmonary infection was induced in the rats as previously described (Bakker-Woudenberg et al., 1979; Kesteman et al., 2010). The trachea of each rat was cannulated under general anesthesia, and the lungs were inoculated with 0.05 mL of a saline suspension of *K. pneumonia* containing 2 × 10^6^ CFU/mL. The control rats were inoculated with saline only. The clinical status of each infected rat was recorded twice daily.

Germfree male Wistar rats (Vital River Laboratory, Beijing, China), aged 5 weeks old, at weights of 180–200 g, and were housed under specific-pathogen-free conditions, with a 12-h light/12-h dark cycle. The experiment followed the Guidelines of Experimental Animal Care issued by the Animal Welfare and Research Ethics Committee at Qingdao Agriculture University.

Rats were divided into four subgroups: (1) Control group (*n* = 30); (2) *K. pneumonia* disease (KPD) model group (*n* = 30): KPD rats were injected with physiologic saline; (3) KPD with injection group (*n* = 30): KPD rats were injected with CEQ injection (12.5 mg/kg). (4) KPD with CEQ-microspheres (*n* = 30): KPD rats were injected with 12.5 mg/kg CEQ-coupled microspheres. After the injection of bacteria for 3 days, treatment was initiated when an animal showed clinical signs of infection (coughing, close-set eyes, immobility, quilted coat, or hunched posture) (Vasseur et al., 2014) and consisted of a tail vein injection of 12.5 mg/kg of CEQ.

#### 2.5.2. Preparation of bronchoalveolar lavage fluid (BALF)

At predetermined time points (0, 4, 12, 24, 48 h) after dosing, rats were anesthetized by intraperitoneal injection of chloral hydrate (300 mg/kg). Four milliliters of sterile physiologic saline was instilled three times *via* the tracheal cannula and recovered by gentle manual aspiration. The three lavage fractions were centrifuged at 2000×*g* for 10 min at 4 °C, and the cell-free supernatants were stored at −70 °C for subsequent cytokine analyses (Xiao et al., 2013). The cell pellet was washed twice with phosphate-buffered saline (PBS) and suspended in 1 mL of RPMI 1640 containing 10% fetal bovine serum and 1% penicillin–streptomycin solution (Gibco, Carlsbad, CA) in six-well tissue culture microplates. The plates were incubated for 2 h at 37 °C in a humidified atmosphere of 5% CO_2_. After the removal of supernatant, adherent cells were suspended in RPMI 1640 at a concentration of 2 × 10^6^ cells/mL for subsequent phagocytosis analyses. Cell viability was checked with the Trypan blue dye and was >96%. Cell purity checked by the Giemsa dye test was >98%.

#### 2.5.3. Bacterial counts

Each rat was treated with bronchoalveolar lavage and then was sacrificed in a CO_2_ chamber and its lungs were aseptically removed and homogenized in 10 mL of normal saline. The homogenates were centrifuged at 2500×*g* for 10 min, and the pellets were suspended in 2.5 mL of normal saline. The counts of *K. pneumonia* organisms were obtained after plating serial dilutions of lung homogenates on Mueller–Hinton agar (Bio-Rad, CA, USA). The colonies were counted after 24 h of incubation at 37 °C. The lowest level of detection was 100 CFU/lung, and the rats were considered to be microbiologically cured if they were found to have counts below this level (Vasseur et al., 2014).

#### 2.5.4. Phagocytosis analyses

The uptake of the neutral red dye by macrophages was measured following the procedure of Long et al. (2005), with the following modifications. The cell suspension (2 × 10^6^ cells/mL, 100 μL/well) was incubated in a 96-well flat-bottomed microtiter plate for 2 h at 37 °C in a 5% CO_2_ atmosphere. After one wash with warm PBS (pH 7.4), 200 μL of 0.1% neutral red (Amresco, Solon, OH, USA) solution in PBS was added. To minimize crystal formation during the neutral red assay, the dye solution was incubated overnight at 37 °C and sterile filtered before use. Following 30 min of incubation of the culture plates at 37 °C, the neutral red solution was aspirated, and each well was carefully rinsed three times with PBS. Finally, the intracellular dye was extracted with 200 μL of a mixture of ethanol and acetic acid (1:1 v/v). The mixtures were evaluated at a wavelength of 550 nm on a Bio-Rad 550 microplate reader (Bio-Rad, CA, USA). The absorbance represented phagocytosis by macrophages.

#### 2.5.5. Cytokine measurement

The levels of TNF-α, IL-1β, IL-8, IL-4, IL-10 and IL-13 in the bronchial lavage supernatants were determined using the corresponding ELISA kits (Nanjing Jiancheng, China) according to the manufactures’ instructions.

#### 2.5.6. Histopathological analysis

After intravenous administration of CEQ microspheres to infected rats at a dose of 12.5 mg/kg CEQ, the left lungs were collected for histopathological analysis as described previously. (Kandemir et al., 2015)

### 2.6. Statistical analysis

Values are presented as mean ± standard deviation (SD). Data were analyzed using SPSS V 15.0 (SPSS Inc., Chicago, IL) and the differences between groups were compared with one-way ANOVA followed by Dunnett’s multiple comparison procedure and the *p* value < .05 was considered as statistically significant.

## 3. Results and discussion

CEQ is unsuitable for *in vivo* use due to its short biological half-life and toxicity (Wang et al., 2014). To alleviate these effects and give sustained delivery of therapeutic agents to the lungs, particulate delivery systems such as liposomes and polymeric nano- and micro-particles have been used (Chi et al., 2015; Zhang et al., 2015; Qu et al., 2017). Biodegradable microspheres have been extensively studied for almost half a century and encapsulation using PLA particles is a successful practice in both clinical and research settings (Khairnar et al., 2014; De Clercq et al., 2016). There are several methods for generating PLA particles including spray drying and phase separation / solvent extraction (Aubert-Pouëssel et al., 2004; Acharya et al., 2009). The latter method is most often used but results in single bulk batches requiring large amounts of reagents, time and labor.

**Figure 1.**
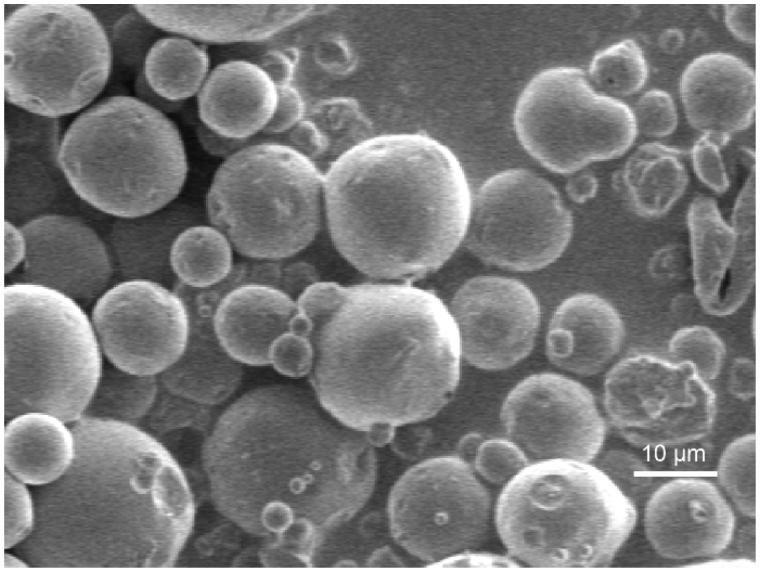
CEQ-loaded PLA microspheres observed by scanning electron microscopy.

We used a spray drying process to overcome these limitations. Our microspheres were globular in appearance and dispersed well (Figure 1). The particle size distribution of the coupled microspheres had mean diameters of 9.8 μm in the ranges of 10% = 4.5 μm, 50% = 9.2 μm and 90% = 15.7 μm. This result demonstrated that it is feasible to use the two-fluid nozzle spray-drying method to prepare CEQ-coupled microspheres in one step without requiring a common solvent for the water insoluble PLA, and the water-soluble CEQ. The average drug loading and encapsulation efficiencies were 18.7 ± 1.2% and 91.6 ± 2.6% (*n* = 3), respectively. Drug-loaded microspheres of highly water-soluble drugs like CEQ using the spray drying technique have given optimal entrapment efficiencies. This is the case because the outer aqueous phase can be rapidly removed by high-speed hot air.

**Figure 2.**
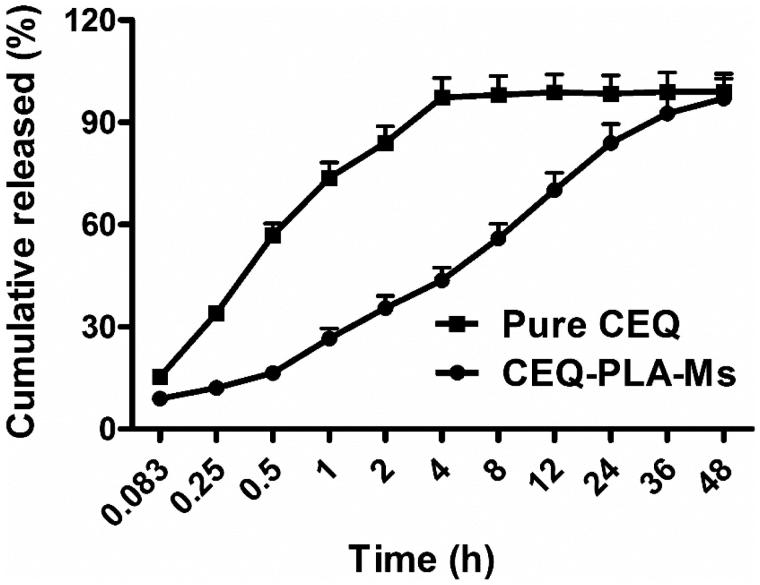
Cumulative CEQ release from CEQ -PLA-microspheres in PBS (pH 7.4). *In vitro* release kinetics were obtained at 37 ± 1 °C by dialysis. CEQ release from the drug stock solution was used as control. CEQ loading was 18.7 ± 1.2%. Data are presented as mean ± SD, *n* = 3.

When we examined *in vitro* CEQ release profiles, the microspheres showed three primary discharge processes. The first was an initial burst of CEQ from the sphere surface (up to 0.5 h). The second was a constant release stage (1–36 h) and the third was a lack of drug release from the microspheres (36–48 h) (Figure 2). Of the total CEQ in the CEQ-PLA-microspheres, 15.2% was released during the first 0.5 h. This reflected CEQ adsorbed on, or incorporated near the microsphere surfaces. In clinical practice, this process would result in rapid treatment by a rapid increase of the active ingredient concentration.

Our microspheres had a relatively low porosity and were degraded slower more than 36 h which thereby extended the release plateau (Freiberg & Zhu, 2004). After 36 h, almost all CEQ was released and this process was much slower than non-incorporated CEQ in solution (Figure 2). Therefore, the *in vitro* performance of the microspheres showed prolonged and sustained CEQ release.

CEQ is widely used in clinical veterinary medicine. However, long-term use of CEQ may result in serious damage to liver or kidney including acute renal failure (Du et al., 2016). Targeting CEQ directly to lung tissues would avoid possible toxic effects to other organs. The size of our microspheres was in the range required for lung targeting so we administered either CEQ-coupled microspheres or CEQ alone *via i.v.* injection to rats (Peppas, 1985). The concentration of CEQ in the kidney was higher than in other tissues after intravenous injection with CEQ solution in rats (Supplement Figure 1(A)). In contrast, the CEQ-PLA-microspheres delivered CEQ primarily to the lung after intravenous injection (Supplement Figure 1(B)). The microspheres showed a combination of lung-targeting and sustained drug release characteristics (Supplement Figure 1), then we conducted pharmacological researches.

Since phagocytosis and elimination of foreign agents are typical functions of macrophages, we examined the effects of CEQ-PLA-microspheres on the phagocytic activity of alveolar macrophages. Failure of this arm of the innate immune response could lead to treatment failure (Taylor et al., 2010).

**Figure 3.**
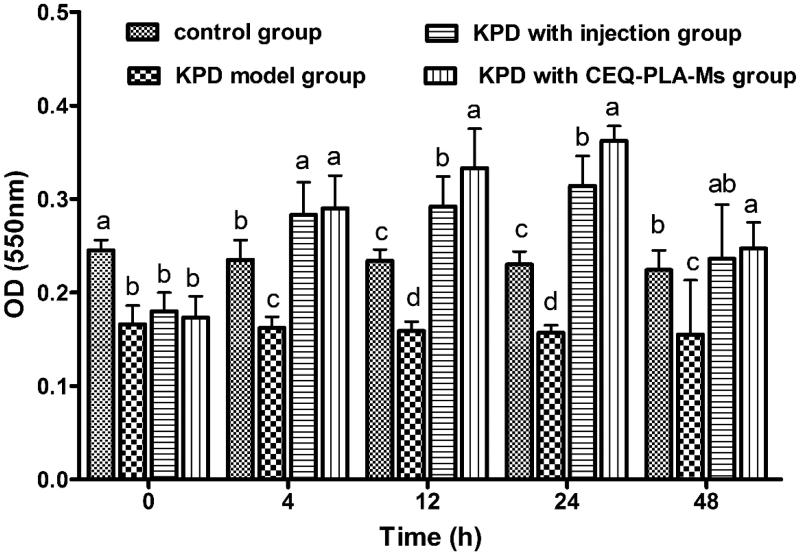
The absorbance at a wavelength of 550 nm represented phagocytosis by macrophages. Data are means ± SD, *n* = 6. ^a–d^Means at that time period for phagocytosis were statistically different (*p* < .05).

The purity of alveolar macrophages exceeds in all groups were exceeding over 98%. The phagocytic activity of alveolar macrophages from the KPD rats was significantly lower than that of the control group. Since the infection itself can cause host immune damage, the bacteria impaired the phagocytic ability of the macrophages. When the rats were challenged with either CEQ-PLA-microspheres or CEQ alone, phagocytic functions were significantly greater than the control untreated and uninfected rats (Figure 3). These results indicated that the CEQ-loaded microspheres provided a successful treatment outcome.

**Figure 4.**
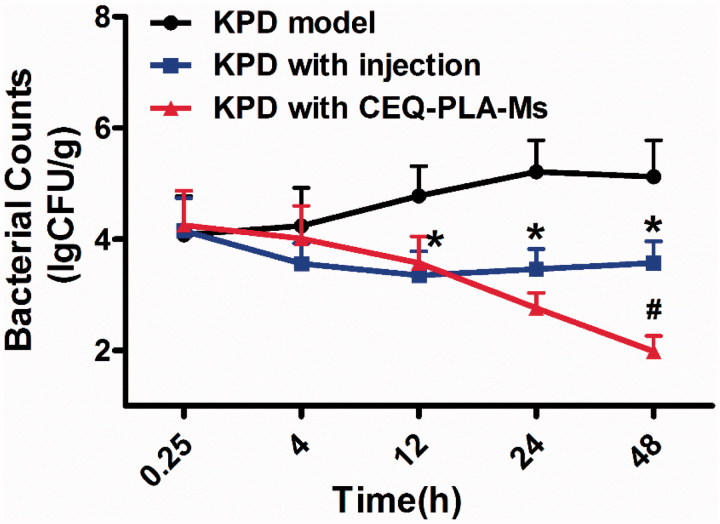
*Klebsiella pneumonia* bacterial counts in lung following *i.v.* administration of a single 12.5 mg/kg dose of CEQ-loaded microspheres or CEQ injection. Each point represents the mean ± SD, *n* = 6. *p < .05 compared with KPD model group. #*p* < .05 compared with CEQ injection group.

To examine the KPD infection model more closely, we determined actual bacterial numbers in the lungs of rats from the different treatment groups. The group lacking treatment displayed a peak of bacterial numbers at 24 h post-infection. After this point, there was a downward trend and the number of bacteria remained at around 10^5^ CFU/g. The group receiving CEQ alone showed a drastic reduction on bacterial numbers as early as 4 h post-infection (Figure 4). However, the rats were not cured of the infection and was most likely due to the short half-life of the drug (*t*_1/2_ = 0.85) (Zhou et al., 2015). The group receiving the CEQ-loaded microspheres achieved a further 1 log reduction of *K. pneumonia*. After 48 h, the rats were considered to be microbiologically cured because the bacterial load was less than 100 CFU/g (Figure 4). In conclusion, we have successfully prepared a drug-loaded microsphere delivery system for the treatment of pneumonia caused by *K. pneumonia.*

**Figure 5.**
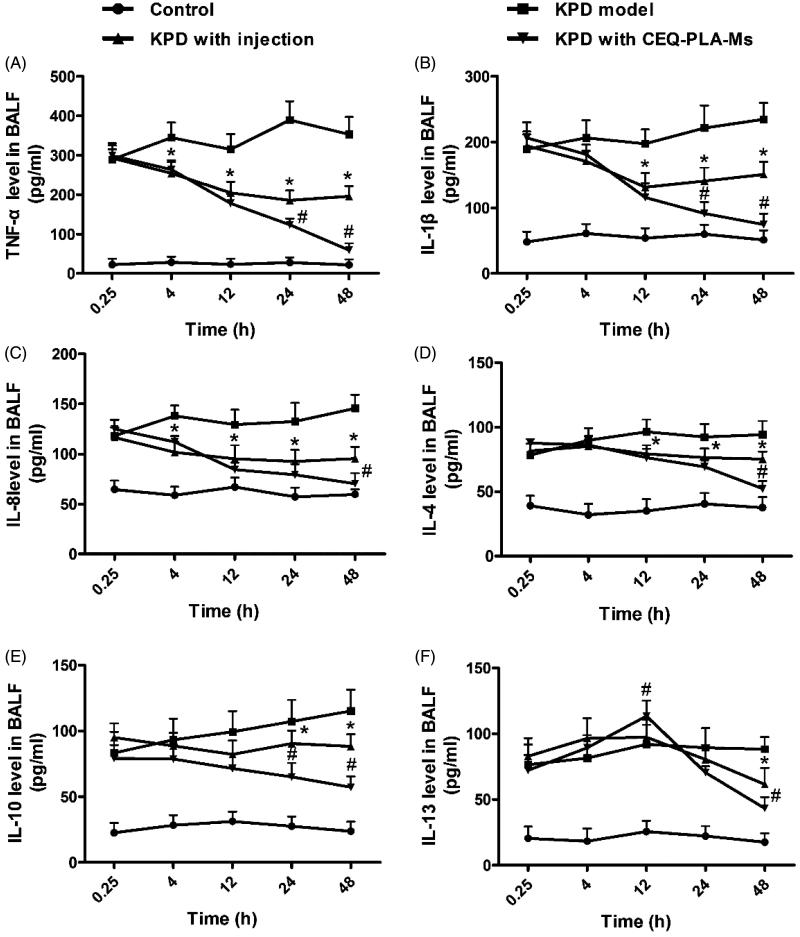
Cytokines in bronchoalveolar lavage fluid. The columns represent the levels of TNF-α (A), IL-1β (B), IL-8 (C), IL-4 (D), IL-10 (E) and IL-13 (F) in four groups of rats: control (no infection or drug), KPD model (infected, no drug), KPD with CEQ injection and KPD with CEQ-loaded PLA-microspheres. Results are given as mean ± SD, *n* = 6. *p < .05, KPD with injection versus KPD model. #*p* < .05, KPD with CEQ-PLA-Ms versus KPD with injection.

Phagocytosis of pathogens by macrophages plays an important role in immune regulation and involves the migration and adhesion of macrophages. Under the influence of chemokines, macrophages are recruited and migrate to inflammatory sites and thus exert their antibacterial effects (Cruijsen et al., 1992). In our infection model we found increased levels of TNF-α, IL-1β and IL-8 after the rats had been infected with *K. pneumonia* (Figure 5(A–C), KPD model group). This indicated that *K. pneumonia* inflammation upregulated neutrophil immunomodulation by promoting the secretion of these cytokines. The peak secretion was early in infection and TNF-α, IL-1β and IL-8 reached their peaks at 24 h post-infection (Figure 5(A–C)).

These proinflammatory molecules can reciprocally stimulate inflammatory cells, including neutrophils and macrophages, to secrete more cytokines and increase phagocytic function. This process enables rapid bacterial clearance and increases lung inflammation (Zamuner et al., 2005). In the previous study, sulfated polysaccharides from *Dendrobium huoshanense* promote macrophage secretion of TNF-α, IL-6 and IL-12 that are beneficial for resisting foreign pathogen invasion (Jiao et al., 2010; Im et al., 2014; Fan et al., 2015). When bacterial numbers decrease, proinflammatory molecules return to normal levels.

In the present study, the anti-inflammatory factors in the CEQ alone and CEQ microsphere groups showed decreasing trends consistent with lower levels of bacteria (Figure 5(A–C)). At the beginning of infection, both proinflammatory and anti-inflammatory factors increased. In both these CEQ treatment groups, IL-4, IL-10 and IL-13 reached their peaks at 12 h post-infection (Figure 5(D–F)). When the bacterial numbers had declined, the anti-inflammatory factors IL-4, IL-10 and IL-13 were inhibited. This resulted in a decrease in inflammatory cells and an inhibition of inflammatory cytokines TNF-α, IL-1β and IL-8 (Figure 5(A–C)).

The start of an infection leads to secretion of proinflammatory cytokines resulting in inflammation that contributes to bacteria removal. Anti-inflammatory factors limit excessive inflammatory development and both these effects are time dependent and when not in synchrony, may result in inflammatory tissue injury. Changes in cytokine levels and types are secondary manifestations of drug bactericidal effects. Therefore, the cytokine changes in the CEQ-loaded microsphere group had the best bactericidal effects (Figure 5).

**Figure 6.**
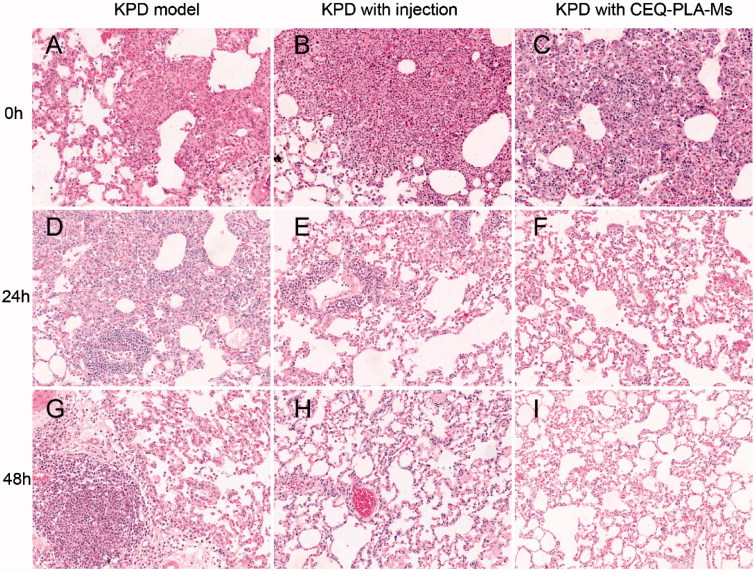
H and E staining of lung tissues from rats after experimental treatments KPD model (infected, no drug) (A, D, and G); KPD model treated with CEQ only injection (B, E and H); KPD model treated with CEQ-PLA-microspheres (C, F and I). Haematoxylin–eosin. Magnification 20×.

We next determined whether the curative effect of CEQ-loaded microspheres was accompanied by tissue destruction. We used the rat infection model and examined lung tissues from the treatment groups by H & E staining. There was a significant infiltration of inflammatory cells into peribronchiolar and perivascular connective tissues in non-treated infected rats. In contrast, CEQ alone and CEQ-PLA-microsphere treatments remarkably attenuated pulmonary inflammation at 12.5 mg/kg. In addition, rat lungs from the CEQ-loaded microsphere group had fewer inflammatory cells than the other group (Figure 6). Thus, the histopathological results were consistent with the bacterial count results.

## 4. Conclusions

This is the first report of the successful use of CEQ-PLA-microspheres as a drug delivery system for clearing experimental *K. pneumonia* lung infections. We prepared CEQ-loaded microspheres using a spray-drying method that had physio-chemical properties and sizes suitable for *in vivo* use. This work adds to the already significant domain of targeted drug delivery systems that hold promising alternatives over conventional methods of drug delivery.

## Supplementary Material

IDRD_Hao_et_al_Supplemental_Content.docx
