# Supplementary material for: Cefquinome-loaded microsphere formulations against Klebsiella pneumonia infection during experimental infections
Source: Drug Deliv. 2018 Apr 13;25(1):909–15. doi: 10.1080/10717544.2018.1461958 (PMC6058672; doi:10.1080/10717544.2018.1461958)
Supplement: IDRD_Hao_et_al_Supplemental_Content.docx [file IDRD_A_1461958_SM9596.docx]

**Supplementary Information**

**Cefquinome-loaded microsphere formulations against Klebsiella pneumonia infection during experimental infections**

Shaoqi Qu,^1,2^ Cunchun Dai,^1,2^ Jiajia Zhu,^1,2^ Li Zhao,^1,2^ Yuwen Li,^1,2^ Zhihui Hao^1,2*^

^1^Agricultural Bio-pharmaceutical Laboratory, Qingdao Agricultural University, Qingdao 266109, P.R. China

^2^National-Local Joint Engineering Laboratory of Agricultural Bio-pharmaceutical Technology, Qingdao 266109, P.R. China

*Corresponding author: Prof. and Dr. Zhihui Hao. Tel: +86 0532 8803 0364; E-mail:  [abplab@126.com.](mailto:275165179@qq.com)

**Supplement Figure 1**


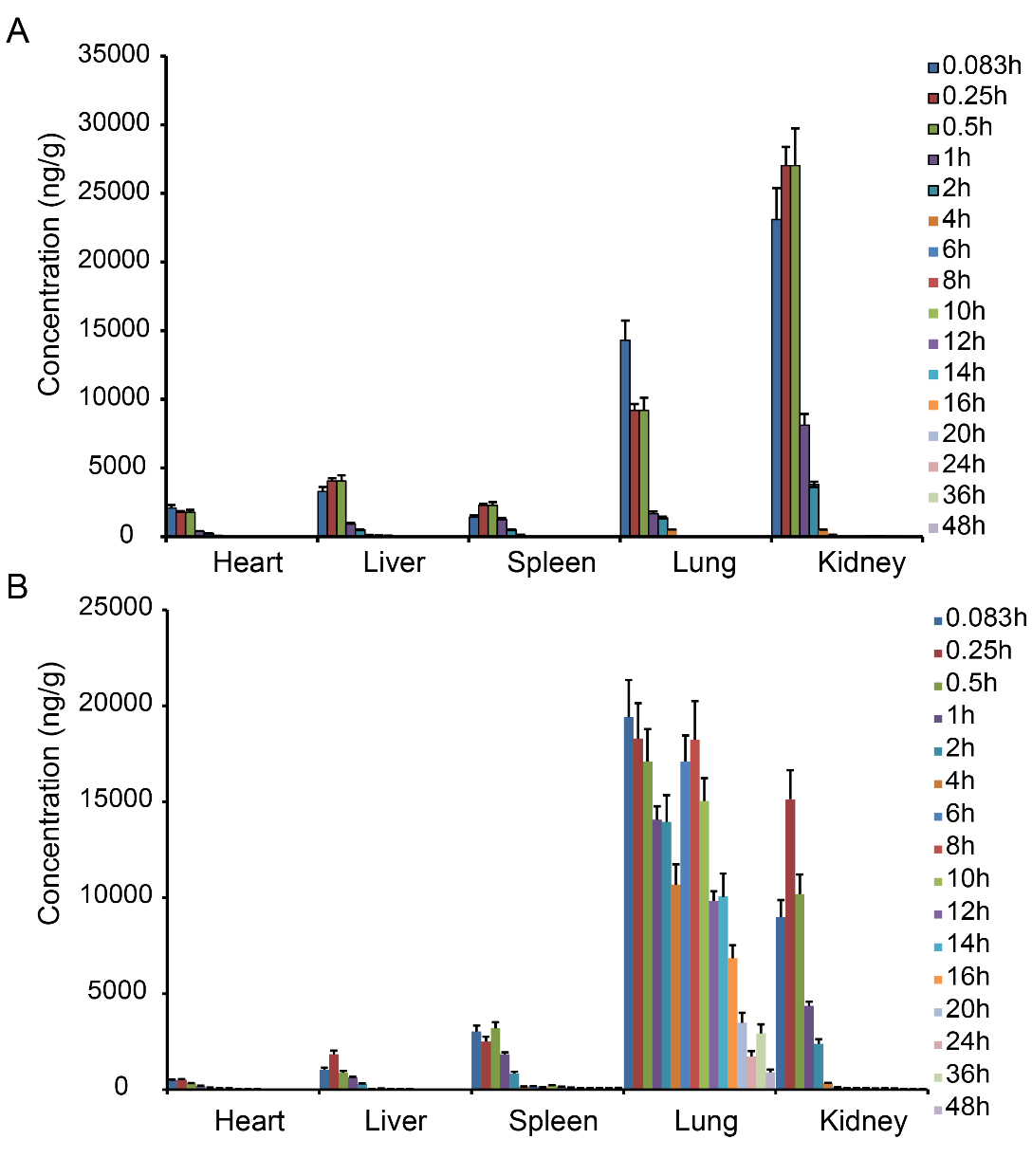


**Figure 1.** (A) Distribution of CEQ in mouse tissues following i.v. administration of a single dose (12.5 mg/kg) of CEQ. Each point represents the mean ± SD from six rats. (B) CEQ distribution in mouse tissues following i.v. administration of a single 12.5 mg/kg dose of CEQ-PLA-microspheres. Each point represents the mean ± SD from six rats.
